# Modeling SARS-CoV-2: Comparative Pathology in Rhesus Macaque and Golden Syrian Hamster Models

**DOI:** 10.1177/01926233211072767

**Published:** 2022-02-05

**Authors:** Shambhunath Choudhary, Isis Kanevsky, Soner Yildiz, Rani S. Sellers, Kena A. Swanson, Tania Franks, Raveen Rathnasinghe, Raquel Munoz-Moreno, Sonia Jangra, Olga Gonzalez, Philip Meade, Timothy Coskran, Jessie Qian, Thomas A. Lanz, Jillian G. Johnson, Cassandra A Tierney, Justin D. Smith, Kristin Tompkins, Arthur Illenberger, Paula Corts, Tara Ciolino, Philip R. Dormitzer, Edward J. Dick, Vinay Shivanna, Shannan Hall-Ursone, Journey Cole, Deepak Kaushal, Jane A. Fontenot, Carles Martinez-Romero, Meagan McMahon, Florian Krammer, Michael Schotsaert, Adolfo García-Sastre

**Affiliations:** 1 Pfizer, Pearl River, New York, USA; 2 Icahn School of Medicine at Mount Sinai, New York, New York, USA; 3 Pfizer, Groton, New York, USA; 4 Texas Biomedical Research Institute, San Antonio, Texas, USA; 5 New Iberia Research Center, New Iberia, Louisiana, USA

**Keywords:** SARS-CoV-2, animal models, rhesus macaque, hamster, pneumonia

## Abstract

Coronavirus disease 2019 (COVID-19) in humans has a wide range of presentations, ranging from asymptomatic or mild symptoms to severe illness. Suitable animal models mimicking varying degrees of clinical disease manifestations could expedite development of therapeutics and vaccines for COVID-19. Here we demonstrate that severe acute respiratory syndrome coronavirus 2 (SARS-CoV-2) infection resulted in subclinical disease in rhesus macaques with mild pneumonia and clinical disease in Syrian hamsters with severe pneumonia. SARS-CoV-2 infection was confirmed by formalin-fixed, paraffin-embedded (FFPE) polymerase chain reaction (PCR), immunohistochemistry, or in situ hybridization. Replicating virus in the lungs was identified using in situ hybridization or virus plaque forming assays. Viral encephalitis, reported in some COVID-19 patients, was identified in one macaque and was confirmed with immunohistochemistry. There was no evidence of encephalitis in hamsters. Severity and distribution of lung inflammation were substantially more in hamsters compared with macaques and exhibited vascular changes and virus-induced cytopathic changes as seen in COVID-19 patients. Neither the hamster nor macaque models demonstrated evidence for multisystemic inflammatory syndrome (MIS). Data presented here demonstrate that macaques may be appropriate for mechanistic studies of mild asymptomatic COVID-19 pneumonia and COVID-19-associated encephalitis, whereas Syrian hamsters may be more suited to study severe COVID-19 pneumonia.

## Introduction

The devastating global impact of severe acute respiratory syndrome coronavirus 2 (SARS-CoV-2) and its associated coronavirus disease 2019 (COVID-19) illness on human health has required rapid development and understanding of animal models for use in assessing the efficacy of potential preventative and therapeutic modalities. The use of these models is expected by regulatory agencies not only to demonstrate efficacy, but, in the case of vaccines, also to demonstrate a lack of vaccine-associated enhanced respiratory disease. Over the past year, numerous reports detailing the susceptibility to and manifestations of SARS-CoV-2 in animal models have been published, which have built on animal model data from the previous coronavirus diseases, SARS in 2002-2003 and Middle East Respiratory Syndrome (MERS) in 2012.^1,2^ These models have included an assortment of both old and new world primates (including Rhesus and Cynomolgus macaques), mice, ferrets, rabbits, cats, and hamsters.^2^ Considerations in selecting models for vaccine development include immune competence, susceptibility to the specific human pathogen, availability of reagents, historical control data, technical experience with the species, and husbandry-related factors (eg, housing). It is also preferable if these models have some clinical, microscopic, or macroscopic endpoints that can be correlated with virus titers. Because most species susceptible to SARS-CoV-2 consistently have respiratory tract infection (as confirmed by viral replication), most efficacy studies evaluating prophylactic (and therapeutic) modalities have focused primarily on respiratory tract endpoints (eg, polymerase chain reaction [PCR] of nasal swabs, bronchoalveolar lavage fluid, and tissues; respiratory tree histology; and microcomputed tomography [microCT] or radiographs). Most SARS-CoV-2 efficacy models have used nonhuman primates, Syrian hamsters, and mice,^3-13^ although other species may be susceptible, such as cats, mink, ferrets, and dogs.^14^ To date, no animal model of multisystemic inflammatory syndrome (MIS) is reported.

COVID-19 presents with respiratory symptoms and other nonrespiratory manifestations most notably fever and gastrointestinal, cardiovascular, and neurological symptoms.^15,16^ Although the range of symptoms for COVID-19 is similar to seasonal influenza, the proportion of severe and critical illnesses are higher with COVID-19. For SARS-CoV-2 infections, data to date suggest that 80% are asymptomatic or cause mild illness, 15% result in severe illness, requiring oxygen, and 5% result in critical illness, requiring ventilation.^17^ Suboptimal type I interferon responses to the virus have been demonstrated in individuals with severe COVID-19, suggesting an important genetic component to susceptibility. These include deficits in Toll-like receptor 3 (TLR3), interferon regulatory factor 7 (IRF7), and interferon regulatory factor 9 (IRF9) signaling as well as the neutralizing autoantibodies to type I interferons, which decrease protective type I interferon responses.^18^

The SARS-CoV-2 spike glycoprotein (S) interacts with its host receptor, angiotensin-converting enzyme 2 (ACE2), to mediate viral cell entry. Cell entry of virus also depends on S priming by host cell proteases such as serine protease transmembrane serine protease 2 (TMPRSS2).^19^ Mouse ACE2 does not bind to wild-type S of all SARS-CoV-2 isolates. While mouse adapted SARS-CoV-2 viruses and genetic modifications for expression of hACE2 in mouse respiratory epithelial cells allow for infection by SARS-CoV-2, these appear to develop highly diverse pathologies after infection, depending on the levels and cell type–specific expression of humanized ACE2 (hACE2).^20^ In natural disease models, animals with endogenous ACE2 compatible with S may develop respiratory infection but often have subclinical disease. There is presently no natural disease model of SARS-CoV-2 that recapitulates the complete spectrum of clinical and pathological findings in humans.

Efficacy data from rhesus macaques with the Pfizer-BNT BNT162b2 mRNA vaccine have been reported previously.^5^ This article serves as a companion to that publication with more details around pathology findings in the macaque model, performed in collaboration with the Southwest National Regional Primate Research Center (SNPRC). In addition, we have included data from the Syrian hamster model of SARS-CoV-2 being developed in collaboration with Icahn School of Medicine at Mount Sinai (ISMMS). Similarities and differences between these models and human COVID are discussed.

## Materials and Methods

### Animal Husbandry

#### Macaques

Male rhesus macaques (*Macaca mulatta*), aged 2 to 7 years, were transferred from the University of Louisiana at Lafayette–New Iberia Research Center (NIRC) to the animal biosafety level 3 facility at SNPRC, Texas Biomedical Research Institute (San Antonio). All studies were performed according to Association for the Assessment and Accreditation of Laboratory Animal Care (AAALAC) International and the National Institutes of Health (NIH) Guide for the Care of Use of Laboratory Animals recommendations under protocols approved by the Texas Biomedical Research Institute Animal Care and Use Committee.

SARS-CoV-2 challenge was performed under tiletamine zolazepam (Telazol; 3 mg/kg) anesthesia as described previously.^5^ Three macaques received 1.05 × 10^6^ plaque forming units (PFU) of the SARS-CoV-2 USA-WA1/2020 isolate, with half of the inoculum administered intranasally (0.25 mL) and the other half intratracheally (0.25 mL). Age- and sex-matched macaques (n = 3) were mock-challenged with Dulbecco’s Modified Eagle Medium (DMEM), supplemented with 10% fetal calf serum (FCS) intranasally (0.25 mL) and intratracheally (0.25 mL; mock-infected animals).

Macaques were monitored by a veterinary clinician for rectal body temperature, weight, and physical examination at 7-, 0-, 3-, and 6-day post-infection (dpi) and at the day of necropsy under general anesthesia. Animals were euthanized (Fatal Plus injection using IV catheter, ≥100 mg/kg) at 7- or 8-dpi for histopathological analyses. All major organ tissues, including the lung (7 sections; 1 sample from each lobe), kidney, liver, spleen, large and small intestine, heart, bone marrow, nasal septum, tongue, trachea, mediastinal lymph node, mucocutaneous junctions, and brain were collected during necropsy and fixed in 10% phosphate-buffered saline (PBS)-buffered formalin.

#### Hamsters

Female Syrian golden hamsters, aged 6 to 8 weeks, were purchased from Envigo and housed in a temperature-controlled environment with 12 hr of light per day at ISMMS (New York, NY). Hamsters were transferred into the Centers for Disease Control and Prevention/U.S. Department of Agriculture (CDC/USDA)-approved biosafety level 3 (BSL-3) facility at the Center for Comparative Medicine and Surgery (CCMS) facility 4 days prior to onset of experiments involving viral infections. Female litter mates were housed in pairs in ventilated cages with ad libitum access to food and water. Environmental enrichment consisted of access to gnawing blocks, extra nesting material, and weekly fresh vegetables. All experimental procedures and protocols were approved by the Institutional Animal Care and Use Committee at ISMMS, in accordance with the institutional and national guidelines and regulations, and performed in AAALAC-certified facilities.

Hamsters were challenged with 0.1 mL of 1 × 10^5^ PFU of SARS-CoV-2 USA-WA1/2020 isolate (virus-infected, n = 12) or sterile PBS (mock-infected, n = 12) by the intranasal route under intraperitoneal ketamine (100 mg/kg) and xylazine (10 mg/kg) anesthesia. Hamsters were monitored by a veterinary clinician for body weight and physical examination for 7 dpi under general anesthesia. Four animals each in the virus-challenged and mock-challenged groups were euthanized (by intraperitoneal injection of sodium pentobarbital ≥150 mg/kg) at 3, 5, and 7 dpi for virological and histopathological analyses. At necropsy, half of the lungs (left lobes), duodenum, kidney, and brain were collected and fixed in 10% PBS-buffered formalin. The remaining halves of the lungs and full nasal turbinates were immediately frozen at −80°C for the viral plaque assay.

### Histopathology

Necropsy, tissue processing, and histology for macaques were performed by SNPRC and for hamsters by ISMMS. Samples were fixed in 10% neutral buffered formalin and processed routinely into paraffin blocks. Tissue blocks were sectioned to 5 μm and stained with hematoxylin and eosin (Cat. No. H9627; Sigma-Aldrich, St. Louis, MO) or Gram stain (Cat. No. AR175; Agilent, Santa Clara, CA) as per manufacturer’s protocol. Microscopic evaluation was performed blindly by SNPRC (macaques), ISMMS (hamsters), and Pfizer (both macaques and hamsters) pathologists and a consensus was reached among pathologists before unblinding.

### Immunohistochemistry (IHC) and In Situ Hybridization (ISH)

Formalin-fixed, paraffin-embedded (FFPE) tissue sections were mounted on SuperFrost Plus slides (Cat. No. 12-550-15; Thermo Fisher Scientific, Waltham, MA) and sectioned to a thickness of 5 µm for both IHC and ISH analysis.

The IHC was performed on the Leica Bond RX (Leica Biosystems, Buffalo Grove, IL) using a mouse monoclonal antibody directed against the SARS-CoV-2 nucleocapsid protein (N) (Cat. No. bsm-49131M; Bioss, Woburn, Massachusetts, clone 8G8A, 1/200 dilution) and a mouse anti-cytomegalovirus (CMV) monoclonal antibody (clone 8B1.2, Cat. No. MAB810R; EMD Millipore, Burlington, MA; 1/400 dilution). Cross-reactivity of anti-CMV antibody to rhesus CMV (rhCMV) was confirmed using an rhCMV-positive control tissue. Tissues sections were pretreated with Epitope Retrieval 2 (Cat. No. AR9640; Leica Biosystems) or Epitope Retrieval 1 (Cat. No. AR9961; Leica Biosystems) for 20 min followed by primary antibody incubation for 30 min and detection with either DAB or Red Leica Refine kits (DS9800 or DS9390; Leica Biosystems). A mouse IgG2b κ isotype control was also tested for nonspecific staining (Cat. No. 557351; BD Biosciences, San Jose, CA). All slides were counterstained with hematoxylin, dehydrated through graded ethanol and xylene, and permanently mounted with coverslips.

ISH was performed using the RNAscope 2.5 LSx Assay—RED reagent kit (Cat. No. 322750; Advanced Cell Diagnostics, ACD, Newark, CA) according to the manufacturer’s guidelines on a Leica Bond Rx (Leica Biosystems) as described previously.^4^ SARS-CoV-2 antisense-specific probe v-nCoV2019-S (Cat. No. 848568; ACD) targeting the positive-sense viral RNA, SARS-CoV-2 sense-specific probe v-nCoV2019-orf1ab-sense (Cat. No. 859158; ACD) targeting the negative-sense genomic viral RNA (indicative of replicating virus), and an *Escherichia coli* DapB probe (Cat. No. 312038; ACD) as negative a control were used. Macaque and hamster tissue quality was assessed, using either a *Homo sapiens* (Cat. No. 313908; ACD) or *Cricetulus griseus* (Cat. No. 450468; ACD) peptidylprolyl isomerase B positive control probe, respectively.

### SARS-CoV-2 Viral RNA Quantification on FFPE Tissue Blocks by Quantitative Real-Time PCR

#### RNA isolation

The FFPE tissue sections, approximately 10 µm thick, were deparaffinized using 1 mL of xylene, vortexed vigorously for 10 sec, and centrifuged at full speed for 2 min. After precipitation of the sample and removal of the supernatant, residual xylene was removed by washing with 1 mL of 100% ethanol, mixed by vortexing, and centrifuged again at full speed for 2 min. The supernatant was then removed, and the samples were incubated at 37°C until all residual ethanol had evaporated. RNA was extracted using the RNeasy FFPE kit (Qiagen, #73504) on Qiacubes according to manufacturer’s instructions. After elution, 1 µL of each sample was evaluated for total RNA concentration using standard 260 nm absorbance on a NanoDrop 1000 (Thermo Scientific) ultraviolet-visible (UV-Vis) spectrophotometer, and the remaining sample was stored at −80°C.

#### Quantitative real-time PCR

Quantification of the SARS-CoV-2 virus N, ORF1ab, and S genes was performed using the TaqPath COVID-19 Combo Kit (Thermo Fisher; Cat. No. A47814). Standard curves were generated by testing SARS-CoV-2 genomic RNA (ATCC, #VR-1986D) in PBS, processed through the extraction procedure. The COVID-19 PCR master mix was prepared by adding 6.25 µL TaqPath 1-Step Multiplex Master Mix (No ROX) (4X), 1.25 µL COVID-19 Real-Time PCR Assay Multiplex, and 12.5 µL RT-PCR grade water. Five microliters of RNA sample or water “no template control” (NTC) was added to the master mix. Real-time PCR was performed on a QuantStudio 12K Flex PCR instrument in a 96-well, 0.1 mL assay plate (Applied Biosystems) in fast mode. The thermal conditions were 25°C for 2 min, 53°C for 10 min, with an initial denaturation step of 95°C for 2 min followed by 40 cycles of 95°C for 3 sec and 60°C for 30 sec. Data were analyzed using the Applied Biosystems QuantStudio 12K flex software. Thresholds were set at 60,000 for the N gene, 100,000 for the S gene, and 250,000 for ORF1ab to determine the cycle threshold (CT) value. Concentration of viral RNA (copies/mL) was interpolated from the extracted standard curve. The cutoff for positivity (lower limit of detection [LLOD]) was established at 300 copies/mL for the N gene and 400 copies/mL for the ORF1ab gene. The LLOD for S gene was not defined to the same rigor as the N and ORF1ab genes in the lab-developed validation package; the S gene data were fitted to the PBS validation standard curve to determine “a reference point,” but the cutoff was based on presence of amplification <37 CT, and the CT cutoff was used in the algorithm to call presence or/absence. Samples were tested in duplicate.

### SARS-CoV-2 Viral Quantification on Hamster Tissues by Plaque Assay

The titer of infectious virus in the hamster tissues was measured by the SARS-CoV-2 plaque assay as described previously.^21^ Briefly, lungs and nasal turbinates were homogenized in 1 mL PBS using a pestle. After brief centrifugation at 13,000 rpm for 10 min, the clarified supernatant was 10-fold serially diluted, starting from 1:10 dilution. Pre-seeded Vero-E6 cells were incubated with diluted lung homogenates for 1 hr at room FFPE and then overlayed with a 1 mL mixture of 2% oxoid agar and 2X MEM supplemented with 2% fetal bovine serum (FBS). After 72 hr of incubation at 37°C, 5% CO2, the plates were fixed in 4% formalin, followed by immune-staining of infected cells with anti-mouse SARS-CoV-2 nucleoprotein and anti-mouse SARS-CoV-2 spike monoclonal antibodies. After incubation with primary antibodies, horseradish peroxidase (HRP)-conjugated anti-mouse secondary antibody was added for 1 hr. The plaques were visualized with TrueBlue substrate (KPL Seracare). The final viral titers were calculated as PFU/mL. The cutoff for positivity (LLOD) was established at 1 plaque for 150 µL tissue homogenates (6.67 plaque/mL).

### RhCMV DNA Quantification on FFPE Tissue Blocks by qPCR

FFPE sections of the brain were deparaffinized as described above, and DNA was extracted using a QIAamp DNA Tissue Kit (Qiagen) according to manufacturer’s instructions. Custom Taqman assays for rhesus used sequences published by Kaur et al^22^: forward primer GTTTAGGGAACCGCCATTCTG, reverse primer GTATCCGCGTTCCAATGCA, and probe TCCAGCCTCCATAGCCGGGAAGG. Rhesus glyceraldehyde 3-phosphate dehydrogenase (GAPDH; Life Technologies RN01775763_g1) was used to confirm detection of genomic DNA. A previously confirmed rhCMV-positive DNA sample was used as a positive control. qPCR was run in triplicate using 50 ng input DNA, Taqman Universal Master Mix II (Life Technologies), and CMV or GAPDH assays in 20 mL reactions on the ABI Viia7 system. Samples were considered negative if the CMV assay did not amplify in the sample, but did amplify in the positive control, and the sample GAPDH amplified properly.

### Statistical Analysis

Weight loss was compared using 2-way analysis of variance (ANOVA). Student’s *t* test was used to determine significant differences in virus titers by plaque assay between the different groups. A *P* value less than .05 was considered statistically significant. All data were graphed, and simple linear regressions were performed in Prism GraphPad (version 8.4.2).

## Results

### Rhesus Macaques

#### Clinical features and lung viral load

SARS-CoV-2-challenged macaques displayed no clinical signs of illness, no elevations in body temperature, and no alterations in body weight compared with the mock-infected animals.

**Figure 1.**
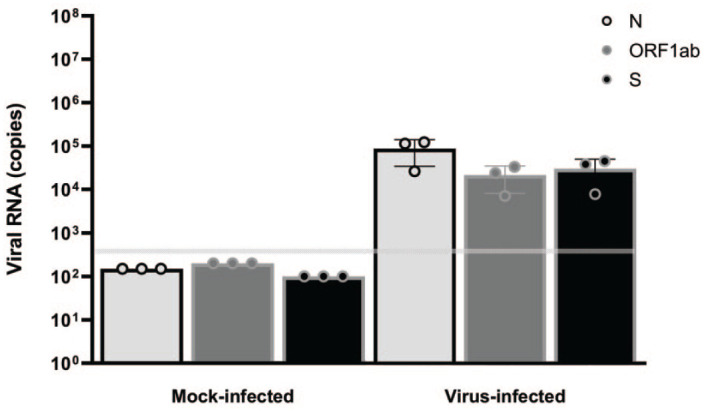
Lung viral loads in rhesus macaques. Male rhesus macaques, 2 to 7 years of age were challenged with 1.05 × 10^6^ total PFU of SARS-CoV-2 (virus-infected, *n* = 3) or with cell culture medium (mock-infected, *n* = 3) split equally between the intranasal and intratracheal routes. Animals were euthanized 7- to 8-dpi. Viral RNA (N, ORF1ab, and S genes) was detected in FFPE lung blocks by real-time polymerase chain reaction. The horizontal gray line indicates the LLOD. Values below the LLOD were set to 1/2 the LLOD. dpi, days post-infection; FFPE, formalin-fixed, paraffin-embedded; LLOD, lower limit of detection; ORF1ab, open reading frame 1ab; PFU, plaque forming units; SARS-CoV-2, severe acute respiratory syndrome coronavirus 2.

We have confirmed previously the kinetics of virus shedding in nasal swabs and bronchoalveolar lavage fluid using RT-qPCR.^5^ In this study, virus infection in the lungs was confirmed by real-time quantitative PCR for SARS-CoV-2 genes (N, ORF1ab, and S) on FFPE samples from seven lung lobes. Viral RNA (N, ORF1ab, and S) was detected in FFPE lung blocks from virus-infected and not mock-infected animals at 7/8-dpi (Figure 1).

#### Pathology

At necropsy (7/8-dpi), macroscopic examination revealed no significant gross pathology observations.

**Figure 2.**
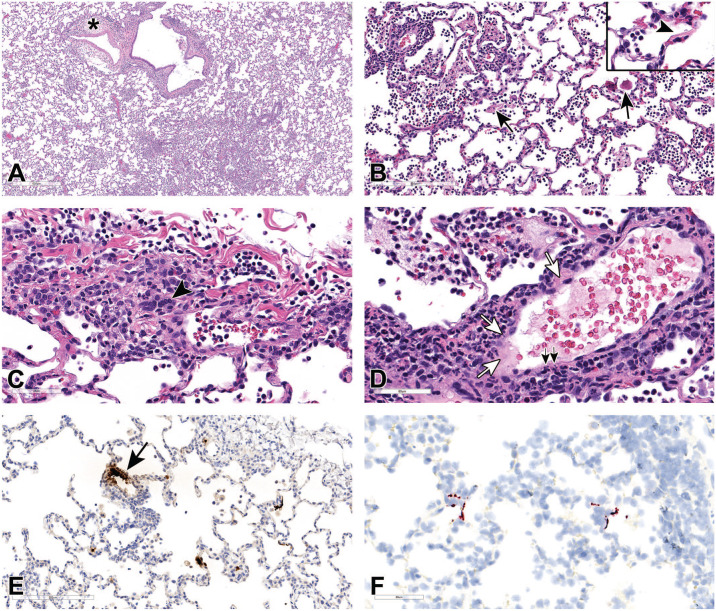
Histopathologic features of SARS-CoV-2 infection in rhesus macaque lungs at 7/8 dpi. (A) Lung sections of virus-infected macaques euthanized at 7/8 days after virus infection showing inflammatory cell infiltrates in the alveolar and peribronchiolar spaces and perivascular spaces with perivascular edema and fibrosis (asterisk). (B) Proteinaceous and fibrinous exudates in alveolar spaces (black arrows) with occasional hyaline membrane lining alveolar surface (arrowhead, inset). (C) Pneumocytes showing cytomegaly, karyomegaly, and multinucleated syncytial features (arrowhead). (D) Affected blood vessels with hypertrophied endothelium, endothelial cell necrosis and loss (black arrows), and vascular wall with edema, inflammatory cells infiltrates, and cell debris (white arrows). H&E, hematoxylin and eosin. (E) and (F) Blood vessel (black arrow) show strong positivity for N (E) and pneumocytes (type I and type II pneumocytes) in the alveolar septa and few macrophages and neutrophils show multifocal strong positivity for N by IHC (E) and for viral RNA by RNAscope ISH using probe that target positive-sense RNA (F). dpi, days post-infection; IHC, immunohistochemistry; ISH, in situ hybridization; SARS-CoV-2, severe acute respiratory syndrome coronavirus 2.

All collected tissues from SARS-CoV-2-infected and control animals were evaluated microscopically. Lungs from SARS-CoV-2-infected macaques had minimal to mild interstitial pneumonia which, when present, affected only 5% to 15% of examined sections (generally in left and right lower lobes). Inflammation typically comprised macrophages and neutrophils with fewer lymphocytes and plasma cells within alveolar, perivascular, and peribronchiolar spaces (Figure 2A). Proteinaceous and fibrinous exudates were observed within alveoli with occasional hyaline membrane formation, indicative of alveolar endothelial damage (Figure 2B). In areas of inflammation, alveolar septae were minimally to mildly thickened by inflammatory cells, edema fluid, and hypertrophic type II pneumocytes; occasionally, type II pneumocyte hyperplasia was present in association with minimal interstitial fibrosis. Cytopathic changes attributed to virus infection were observed in pneumocytes characterized by cytomegaly and karyomegaly, and binucleated or multinucleated giant cells (syncytia; Figure 2C). The endothelial cells of nearby blood vessels were hypertrophied or necrotic, with loss of endothelial lining and occasionally neutrophils-, macrophages-, and lymphocytes-infiltrated edematous vessel walls containing cellular debris, suggestive of vasculitis and endotheliitis (Figure 2D).

The alveolar epithelial cells and a small number of inflammatory cells (macrophages and neutrophils) were immunoreactive for N by IHC (Figure 2E) and for SARS-CoV-2 viral RNA by ISH (Figure 2F) in regions of inflammation. Blood vessels in the region of inflammation were also immunoreactive by IHC (Figure 2E). However, no labeling was observed by ISH for negative-sense genomic viral RNA (data not shown), indicating no detectable viral replication in lung tissue at the time of sample collection.

**Figure 3.**
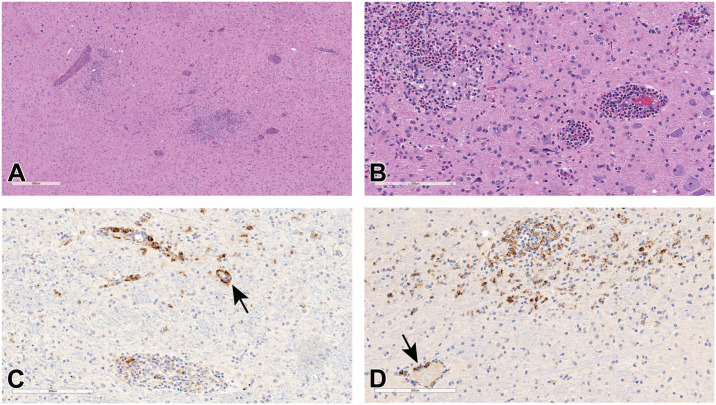
Histopathologic features of SARS-CoV-2 infection in rhesus macaque brain. (A) Brain section from a virus-infected macaque showing inflammatory cell infiltrates within the neuropil and around blood vessels. H&E. (B) Higher magnification showing vacuolated neuropil and blood vessels with hypertrophied endothelium and inflammatory cells in the wall and attached to endothelium. H&E. (C) and (D) Glial cells, neurons, inflammatory cells, and blood vessel (black arrow) showing multifocal strong positivity for N by IHC. H&E, hematoxylin and eosin. IHC, immunohistochemistry; SARS-CoV-2, severe acute respiratory syndrome coronavirus 2.

Among extrapulmonary tissues examined, encephalitis was evident in the brain of one virus-infected animal. Inflammatory cell infiltrates mainly comprised neutrophils with lesser numbers of macrophages, and lymphocytes, which multifocally infiltrated the neuropil and cuffed the blood vessels (Figure 3A). The adjacent neuropil was rarefied, and blood vessels in the affected areas contained hypertrophied endothelium with inflammatory cells attached to endothelium or within the vessel walls (Figure 3B). Glial cells, rare neurons, inflammatory cells, and blood vessels in areas of inflammation were immunoreactive for N by IHC (Figure 3C and D). However, no viral RNA was detected by ISH or PCR assays (data not shown). RNA is less stable and degrades earlier than the protein in FFPE tissue. The brain has lower susceptibility to viral infection and replication as compared with the lungs, possibly due to lower expression of viral receptor ACE2 and cofactor TMPRSS2.^19^ Both lower susceptibility to viral infection/replication and rapid RNA degradation might have contributed to low concentration of viral RNA in the brain tissues to be detected by ISH or PCR.

RhCMV is a common cause of encephalitis in rhesus macaques.^23^ The area of inflammation in the brain was not immunoreactive for rhCMV by IHC and PCR (data not shown), suggesting encephalitis in this animal was not associated with rhCVM infection. Although it’s rare, spontaneous encephalitis in captive rhesus macaques can also be caused by bacterial infection. Review of the 36-year SNPRC pathology database revealed a total of 9 cases of encephalitis out of 2850 rhesus macaque necropsies. Eight out of 9 animals were infants and 7 of these cases were associated with generalized bacterial infection (6 infants and 1 adult); encephalitis in two of the infants was of undetermined etiology. In this study, however, no evidence of bacterial infection was found during routine physical examination or serum biochemistry profile/hematology parameters analysis before or at the start of study. Gross pathology or microscopic lesions often associated with bacterial infection (eg, endocarditis or abscess) were not observed. In addition, no bacteria were identified in tissue sections by H&E or Gram stain (data not shown).

No virus-associated histopathological changes were observed in the other organs examined, including kidney, liver, spleen, large and small intestine, heart, bone marrow, nasal septum, tongue, trachea, mediastinal lymph node, and mucocutaneous junctions. There was no evidence of myocardial injury or inflammation suggestive of the MIS that has been reported in humans infected by SARS-CoV-2.

### Hamsters

#### Clinical features and viral load

**Figure 4.**
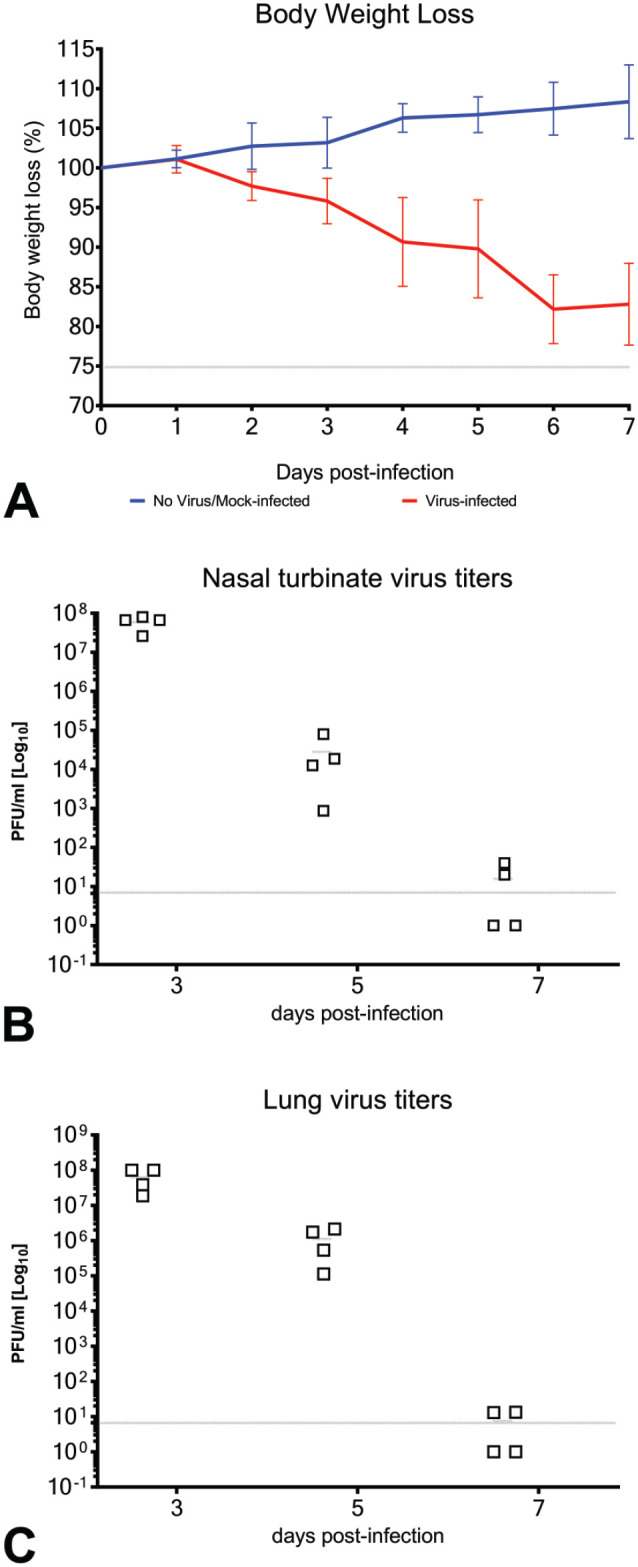
Clinical disease after SARS-CoV-2 infection in hamsters. (A) Body weight changes of SARS-CoV-2-infected and mock-infected hamsters from 0 dpi to 7 dpi. Four animals in each of the treatment groups were euthanized at 3, 5, and 7 dpi. (B) and (C) Quantitation of virus titer by plaque forming assay in the nasal turbinates (B) and lungs (C) of SARS-CoV-2-challenged hamsters at 3 dpi, 5 dpi, and 7 dpi (n = 4/day). Error bars represent SDs of the mean. The horizontal gray lines indicate the LLODs. dpi, days post-infection; LLOD, lower limit of detection; SARS-CoV-2, severe acute respiratory syndrome coronavirus 2.

SARS-CoV-2-infected, but not mock-infected, hamsters exhibited progressive mean body weight loss of up to approximately 15% from 1 to 7 dpi (Figure 4A). Hamsters developed lethargy, ruffled fur, and rapid breathing. No study animals succumbed to disease.

Viral load (SARS-CoV-2 plaque assay) confirmed virus infection and replication in the upper respiratory tract (nasal turbinates) and lower respiratory tract (lungs) 3-, 5-, and 7-dpi. The mean viral loads were comparable across time points in the nasal turbinates (Figure 4B) and in lungs (Figure 4C). The viral loads were highest at 3 dpi and progressively decreased in both upper and lower respiratory tracts from 3 dpi to 7 dpi. At 7 dpi, viral loads in both the nasal turbinates and the lungs were close to or below the assay LLOD.

#### Pathology

At necropsy, lung lobes of challenged animals were edematous and multifocally mottled dark red at 3 and 5 dpi. Challenged animals necropsied at 5 and 7 dpi also showed multifocal areas of macroscopic dark red firm lung lobes (data not shown).

**Figure 5.**
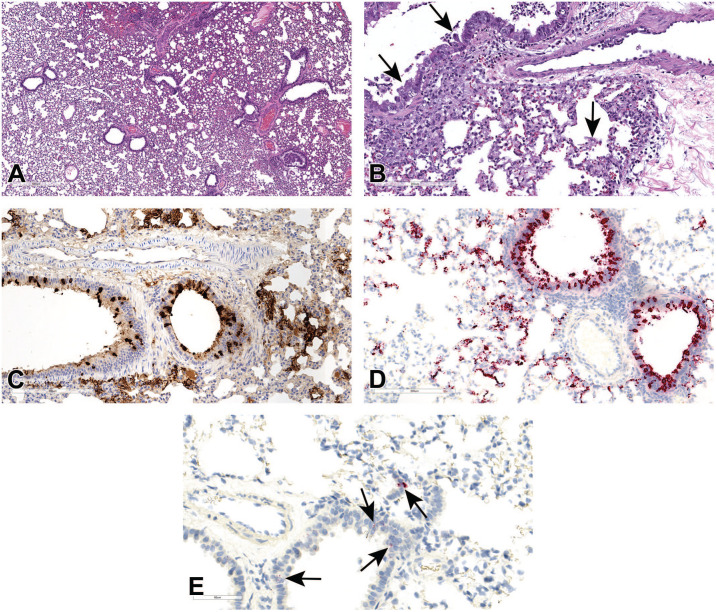
Histopathologic features of SARS-CoV-2 infection in hamster lungs at 3 dpi. (A) and (B) Broncho-interstitial pneumonia at 3 dpi, H&E stained. The interstitium is expanded with inflammatory cell infiltrates and fluid exudate (A). The alveolar and bronchiolar epithelium is multifocally necrotic (black arrows), and the bronchiolar lumen is filled with cellular debris (B). (C) to (E) Bronchiolar and alveolar epithelial cells (pneumocytes) show multifocal strong positivity for N by IHC (C), for viral RNA by RNAscope ISH using probes that target the positive-sense viral RNA (D), and the negative-sense genomic viral RNA (E); arrows point to infected cells containing the rarer negative-sense genomic viral RNA (E). dpi, days post-infection; H&E, hematoxylin and eosin; IHC, immunohistochemistry; ISH, in situ hybridization; SARS-CoV-2, severe acute respiratory syndrome coronavirus 2.

Tissues from SARS-CoV-2-infected and mock-infected animals were microscopically evaluated at 3, 5, and 7 dpi. At 3 dpi, infection was associated with mild to moderate interstitial pneumonia, affecting up to 20% to 30% of the evaluated sections. Alveolar walls were minimally to mildly thickened by congestion and edema, mononuclear inflammatory cell infiltrates, and fibrin; within alveoli, there was multifocal hemorrhage, neutrophilic and monocytic infiltrates, and nuclear and cellular debris (Figure 5A). Alveolar and bronchial epithelia were frequently necrotic, and large and small airways contained variable amounts of cellular debris (Figure 5B). Vascular changes were minimal and included endothelial hypertrophy and occasional transmigration of inflammatory cells, such as neutrophils, lymphocytes, and macrophages through the vascular walls of pulmonary vessels. The bronchiolar and alveolar epithelial cells, but not blood vessels, were immunoreactive for N by IHC (Figure 5C) and for viral RNA by ISH (Figure 5D) in regions of inflammation. Both positive-sense (Figure 5D) and negative-sense viral RNA (Figure 5E) were observed by ISH, suggesting viral replication in lung tissue.

**Figure 6.**
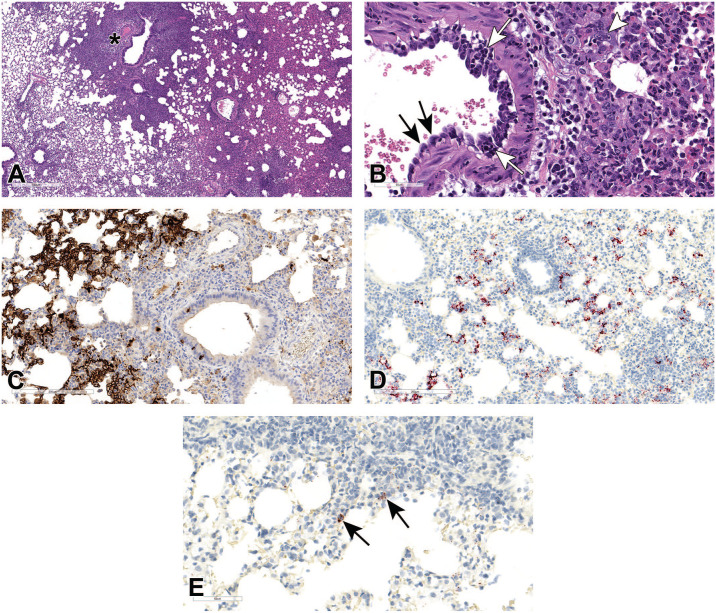
Histopathologic features of SARS-CoV-2 infection in hamster lungs at 5 dpi. (A) and (B) Histopathological changes in the lungs at 5 dpi, H&E stained. Low magnification showing large areas of broncho-interstitial pneumonia caused by inflammation and exudate fluid accumulation with intense peribronchiolar infiltration and perivascular edema (asterisk) (A). High magnification showing cytomegaly, karyomegaly, and syncytia (arrowhead) affected blood vessels with hypertrophied endothelium (black arrows), inflammatory cells infiltrating damaged endothelium and subendothelial space (white arrows), vessel walls containing inflammatory cells and cell debris, and perivascular space expanded by edema and fibrosis (B). (C) to (E) Alveolar epithelium (pneumocytes) shows multifocal strong positivity for N by IHC (C), for viral RNA by RNAscope ISH using probes that target the positive-sense viral RNA (D), and the negative-sense genomic viral RNA (E); arrows point to infected cells containing the rarer negative-sense genomic viral RNA (E). dpi, days post-infection; H&E, hematoxylin and eosin; IHC, immunohistochemistry; ISH, in situ hybridization; SARS-CoV-2, severe acute respiratory syndrome coronavirus 2.

At 5 dpi, increasing broncho-interstitial pneumonia was seen, with many more inflammatory cells around bronchioles, and inflammatory cell infiltrates (predominantly macrophages and lymphocytes with fewer neutrophils), fluid, and hemorrhage within alveolar spaces. The area of lung affected was notably greater, with involvement of approximately 50% to 60% of evaluated lung sections (Figure 6A). Mild to moderate bronchiolar epithelia hyperplasia and alveolar epithelial hyperplasia were present in association with minimal interstitial fibrosis. Cytopathic changes due to virus were evident in bronchiolar epithelial cells, type II pneumocytes, and macrophages, and included multifocal karyomegaly and cytomegaly with scattered bi- or multinucleated (syncytia) cells (Figure 6B). Some intracytoplasmic or intranuclear inclusions appeared to be present in these cells. The endothelial cells of nearby vessels were hypertrophied and often separated from the underlying basement membrane; inflammatory cells were attached to damaged endothelium, accumulated within the subendothelial space, and infiltrated the vessel walls containing cellular debris, indicative of endotheliitis and vasculitis (Figure 6B). Perivascular edema and fibrosis was also observed multifocally in the areas of inflammation (Figure 6A and B). IHC labeling for N (Figure 6C), positive-sense viral RNA (Figure 6D), and negative-sense viral RNA (Figure 6E) were primarily observed in alveolar epithelial cells.

**Figure 7.**
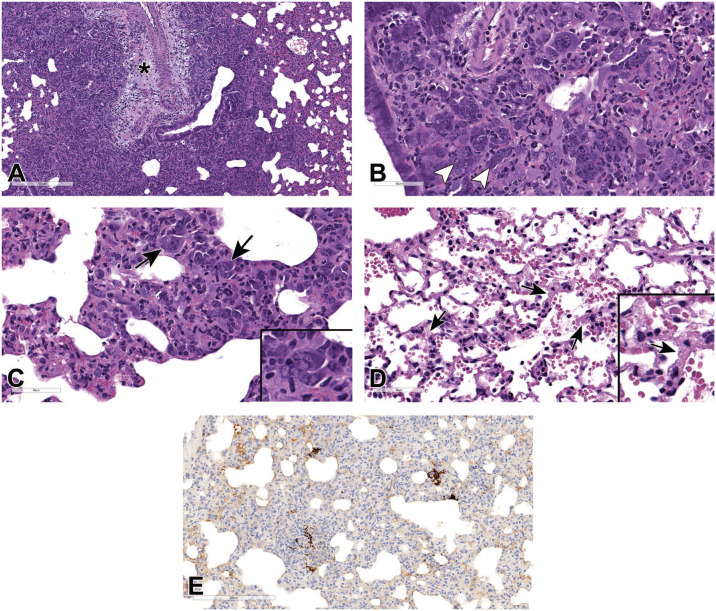
Histopathologic features of SARS-CoV-2 infection in hamster lungs at 7 dpi. (A) to (C) Histopathological changes in the lungs at 7 dpi, H&E stained. Marked increases in lung cellularity caused by proliferation of bronchiolar and alveolar type II pneumocytes and perivascular edema and fibrosis (asterisk) (A). High magnification showing (B) hyperplastic bronchiolar epithelium and adjacent hypercellular lung parenchyma with syncytia (white arrowheads). High magnification showing (C) karyomegalic cells with suspected intracytoplasmic inclusion (black arrows and inset). (D) Black arrows and inset showing hyaline membrane lining alveolar surface. (E) Alveolar epithelium (pneumocytes) shows positivity for N by IHC. dpi, days post-infection; H&E, hematoxylin and eosin; IHC, immunohistochemistry; SARS-CoV-2, severe acute respiratory syndrome coronavirus 2.

By 7 dpi, there was marked broncho-interstitial pneumonia with marked increases in lung cellularity (Figure 7A). The inflammatory cell infiltrates and exudate identified at 3 and 5 dpi were largely replaced by proliferative bronchiolar and alveolar epithelia of bronchioles and mild interstitial fibrosis. There was extensive type II pneumocyte hypertrophy and hyperplasia, which formed irregular and multifocal aggregates (Figure 7A). Perivascular edema and fibrosis were still prominent (Figure 7A) and intact/desquamated type II pneumocytes or macrophages with cytopathic changes (as previously described) due to virus were frequently observed at this stage (Figure 7B and C). Occasionally, hyaline membranes were observed closely adherent to alveolar surface (Figure 7D). IHC labeling for N (Figure 7E) was much less at 7 dpi than at 3 and 5 dpi, and present only in scattered pneumocytes. There was no positive labeling for SARS-CoV-2 RNA evident by ISH (data not shown).

No histopathological changes were observed in other organs collected during necropsy.

## Discussion

We evaluated the rhesus macaque and Syrian hamster models of SARS-CoV-2 infection. In rhesus macaques, SARS-CoV-2 infection was not associated with clinical signs of illness. Microscopic findings were limited to mild multifocal pulmonary inflammation that was localized primarily to the right and left lower lung lobes and, in one case, mild encephalitis. Virus in the affected tissues was confirmed by IHC for N and by ISH or qRT-PCR for viral RNA. There were no clinical signs or macroscopic or microscopic findings suggestive of MIS or myocarditis, which have been seen in humans infected with SARS-CoV-2. In contrast, SARS-CoV-2 infection in Syrian hamsters resulted in decreased body weight and dyspnea. Macroscopic and microscopic findings were limited to the lungs. The severity of lung lesions progressed from mild to moderate at day 3 to severe at day 7. This differed from viral load kinetics that gradually decreased over the course of the study. Comparative pathology findings in macaque and hamster models are outlined in Table 1.

**Table 1.** Comparative COVID-19-Related Pathology Findings Between Macaque and Hamster Models at 7 dpi.

Finding	Rhesus macaque	Hamster
Mean body weight loss	Absent	Present
Pulmonary hyaline membrane formation	Minimal (2/3)	Minimal (2/4)
Bronchial and alveolar epithelium hyperplasia	Mild (3/3)	Marked (4/4)
Cytopathic changes (cytomegaly, karyomegaly, and syncytia formation)	Minimal (3/3)	Moderate (4/4)
Interstitial fibrosis	Minimal (3/3)	Mild (4/4)
Vascular changes (vasculitis and endotheliitis)	Minimal (3/3)	Mild (4/4)
Encephalitis	Mild (1/3)	Absent (0/4)
Blood vessels immunoreactivity to N by IHC in the brain and lungs	Present (Brain 1/3, lungs 2/3)	Absent (Brain 0/4, lungs 0/4)

Numbers in parentheses show number of animals with finding versus number of animals examined.

Abbreviations: dpi, days post-infection; COVID-19, coronavirus disease 201; IHC, immunohistochemistry.

Our finding of mild COVID-19 in rhesus macaques without clinical signs, such as fever, respiratory distress, weight loss, or mortality, is consistent with previous reports using similar infectious challenge doses.^4,5,8^ We confirmed SARS-CoV-2 infection by FFPE-PCR, IHC, and RNAscope ISH (positive-sense viral RNA), without active viral replication (negative ISH for negative-sense genomic viral RNA) in the lung tissue at 7/8 dpi. Chandrashekar et al^4^ demonstrated viral replication in the macaque lung 2 dpi. Singh et al^8^ found that, despite viral RNA persistence in the lungs of immunocompetent macaques, viral replication was not evident at later time points (12-14 dpi). These findings indicate that macaques control SARS-CoV-2 infection and replication, thus avoiding serious lung pathology and clinical signs of disease. However, in contrast, Lu et al^6^ demonstrated that macaques infected with SARS-CoV-2 developed clinical signs of disease, including fever and body weight loss. This difference from our results and others’ may be attributable to the higher viral challenge dose and dose volume used for inoculation. Lu et al^6^ inoculated a total of 4.75 mL of 10^6^ PFU/mL virus, compared with doses of 1 to 2 mL (105-10^6^ PFU/mL) virus in other studies in which macaques were reported to develop limited clinical disease. In addition, the number of routes used for virus inoculation may also influence the severity of clinical disease in the macaque model. Clinical disease was slightly higher (mild to moderate) in the macaques when virus challenge was performed using multiple routes (ocular, intratracheal, and intranasal),^3,8,24^ compared with mild disease observed in studies with only 2 routes of infection (intratracheal and intranasal).^4,5^ Conjunctival administration has been shown to be a potential route of SARS-CoV-2 entry resulting in mild COVID-19 pneumonia in rhesus macaques,^25^ and this may reflect drainage to the respiratory tract through nasolacrimal duct. Additional routes of administration and a higher dose and dose volumes result in higher virus exposure overall, as well as potentially wider distribution deeper into the lung, resulting in more severe lung pathology (macroscopic areas of consolidation correlating with mild-to-moderate, interstitial pneumonia microscopically) and clinical signs of disease.^3,24^ Other factors that contribute to disease severity in nonhuman primate (NHP) are species and age. Baboons are reported to have more severe lung pathology than rhesus macaques or marmosets.^8^ Aged rhesus macaques (15 years old) are reported to develop more severe pneumonia and higher viral titers after SARS-CoV-2 challenge than younger animals.^7^ The macaques used in our studies were young (2-7 years old) and developed mild disease. Furthermore, other factors that could contribute to disease severity after experimental challenge include environmental factors, such as housing (eg, indoor, outdoor, and group) and the source of the NHP (eg, bred in captivity vs. in the wild and the region of origin).

Unlike in the macaque model, our data in the hamster model demonstrated that SARS-CoV-2 replicates efficiently in both upper and lower respiratory tracts and causes severe pathological lesions in the lungs. Consistent with previous studies using Syrian hamsters,^12^ we observed that broncho-interstitial pneumonia continued to escalate and correlated with continued body weight loss from 3 dpi to 7 dpi in animals infected with SARS-CoV-2. In contrast with the kinetics of pneumonia, SARS-CoV-2 replication and SARS-CoV-2 RNA and protein expression in lung were highest on day 3 and diminished by day 5, with minimal viral RNA or protein detected by day 7. As in rhesus macaques, viral replication was not evident at 7 dpi. Pulmonary inflammation and clinical disease in hamsters continued to increase even as replicating virus decreased, similar to that observed in humans with severe COVID-19.^26^

The severity of disease in hamsters compared with macaques may be in part impacted by both susceptibility to virus and virus dose relative to lung volume. Hamsters are highly susceptible to SARS-CoV-2, with as few as 5 infectious particles inoculated intranasally resulting in infection.^27^ Hamsters are also administered a relatively higher dose of virus than nonhuman primates, which may also impact disease severity. The typical inoculum administered to Syrian Hamsters is ~10^3^ to 10^5^ PFU, with many studies administering 10^5^ PFU. The inoculum administered to macaques is not notably higher, typically on the order of ~10^5^ to 10^6^ PFU. Hamsters administered doses of 10^5^ PFU intranasally have more notable weight loss than hamsters administered doses of 10^3^ pfu.^13,27^ Higher doses are also associated with more severe lung CT findings as well as inflammation at day 3 in hamsters administered 10^5^ pfu than 10^3^ pfu.^13,27^ Using estimates of lung volume, the adult male rhesus macaque has a lung volume of ~250× that of the adult hamster.^28,29^ Therefore, in many studies, hamsters are administered a much higher dose of virus on a PFU/lung volume ratio.

Apart from inflammation, we observed characteristic virus-induced cytopathic effects and vascular changes in both macaque and hamster lungs. Changes were more prominent in hamsters than macaques, especially at later time points (day 5 and day 7). Hyperplastic pneumocytes with viral cytopathic-like effects such as cytomegaly, karyomegaly, intracytoplasmic, or intranuclear inclusions are observed in the lungs of COVID-19 patients.^30^ Syncytia formation is a widely reported cytopathic effect associated with SARS-CoV-2 infection in various animal models including cynomolgus macaques and hamsters.^9,10^ Although the viral and cellular mechanisms regulating the formation of these syncytia are not well understood, syncytia formation is thought to be triggered by infected cell surface SARS-CoV-2 S, which causes fusion with ACE2-positive neighboring cells.^31^

Vascular changes associated with SARS-CoV-2 infection include endothelial dysfunction resulting in vasculitis and thrombosis leading to multiple organ dysfunction.^32-34^ We demonstrated vascular alterations such as endothelial cell hypertrophy, endotheliitis, and vasculitis with perivascular edema and inflammation in both models; these changes were more prominent in the hamster model. ACE2 is expressed by endothelial cells and hence can be directly targeted by SARS-CoV-2.^32^ SARS-CoV-2 protein within vessels in areas of inflammation was detected in both the lungs and brain in macaques, but not in hamsters. Syrian hamsters lack the expression of ACE2 in endothelial and smooth muscle cells of pulmonary vessel walls.^35^ This finding indicates that vascular lesions in hamsters are not the direct consequence of viral infection of endothelial cells but likely represent the consequence of immune-mediated effects from infiltrating immune cells. Allnoch et al^36^ recently showed that the circumferential loss of aquaporin 1 is associated with disruption of vascular endothelium in hamsters and may contribute to the loss of intercellular junctions, leading to perivascular edema and other vascular changes in SARS-CoV-2 infection.

We demonstrated SARS-CoV-2-associated encephalitis in one of three macaques and none of the mock-infected animals. The encephalitis was not associated with any CNS-related clinical signs. SARS-CoV-2-associated nervous system involvement is reported in humans and in ACE2-transgenic murine models.^37-39^ In humans, COVID-19-associated brain pathologies, such as encephalitis, cerebral microbleeds, stroke, and diffuse leukoencephalopathy are described, and it is hypothesized that inflammation of blood vessels secondary to virus binding of endothelium (rich in ACE2) results in subsequent breaks and leaks.^37,38^ Another potential route for viral entry into the brain following intranasal inoculation could be through the olfactory nerve that provides a pathway between the olfactory bulb, located near the nasal cavity, and the brain; however, a recent review suggests a weak case for infection through this route.^40^ There are little data on SARS-CoV-2-related encephalitis in nonhuman primates. A few studies reported SARS-CoV-2 infection in macaque brains by PCR. SARS-CoV-2-related brain lesions are not described in detail and, when reported, a viral etiology in the areas of inflammation is not confirmed.^40-42^ In the study described here, vascular changes in the brain, including endothelial cell hypertrophy, endotheliitis, and vascular inflammation, were observed, indicating that the blood-brain barrier may have been disturbed. In addition, the presence of SARS-CoV-2 was confirmed by positive IHC labeling of N in these lesions, making SARS-CoV-2 the most likely etiology in this case, especially given the lack of evidence for other more common causes of encephalitis in rhesus macaques (rhCMV and bacteria). Glial cells and neurons are reported to express ACE2, which renders them potential targets for SARS-CoV-2 infection.^43^

Unlike in the case of one macaque, there were no microscopic findings suggestive of viral infection in the hamster brain; IHC and in situ hybridization were also negative. Imai et al^13^ demonstrated that SARS-CoV-2 can replicate in the brain or olfactory bulb of hamsters by plaque formation assay; however, they could not detect viral antigens or virus-associated brain pathology. Similar to pulmonary blood vessels, lack of ACE2 expression in endothelial cells in the brain might limit hematogenous viral entry into the brain.^35^

In summary, SARS-CoV-2 infection in our rhesus macaques and hamsters recapitulates a range of illness observed in human COVID-19. At the virus dose administered, macaques manifest subclinical COVID-19, whereas the Syrian hamster develops clinical disease and more severe pulmonary lesions. Despite high viral inoculate in the hamster and severe disease, there was no evidence of encephalitis, which may be due to lack of ACE2 expression in the brain vasculature. In contrast, one rhesus macaque developed subclinical encephalitis. Larger studies with extensive evaluation of the brain may help establish the rhesus macaque as a suitable animal model for COVID-19-related neurological disorders in humans.
